# Exposure to the COVID-19 news on social media and consequent psychological distress and potential behavioral change

**DOI:** 10.1038/s41598-023-42459-6

**Published:** 2023-09-14

**Authors:** Ali Montazeri, Samira Mohammadi, Parisa M.Hesari, Hossein Yarmohammadi, Mehdi Rafiei Bahabadi, Fatemeh Naghizadeh Moghari, Farzaneh Maftoon, Mahmoud Tavousi, Hedyeh Riazi

**Affiliations:** 1 Health Metrics Research Centre, Iranian Institute for Health Sciences Research, ACECR, Tehran, Iran; 2 Faculty of Humanity Sciences, University of Science and Culture, Tehran, Iran; 3 Department of Epidemiology and Biostatistics, Schulich School of Medicine and Dentistry, Western University, London, Canada; 4 Iranian Students’ Polling Agency (ISPA), Tehran, Iran; 5 School of Nursing and Midwifery, Shahid Beheshti University of Medical Sciences, Tehran, Iran

**Keywords:** Psychology, Human behaviour

## Abstract

Exposure to coronavirus disease 2019 (COVID-19) news pandemic is inevitable. This study aimed to explore the association between exposure to COVID-19 news on social media and feeling of anxiety, fear, and potential opportunities for behavioral change among Iranians. A telephone-based survey was carried out in 2020. Adults aged 18 years and above were randomly selected. A self-designed questionnaire was administered to collect information on demographic variables and questions to address exposure to news and psychological and behavioral responses regarding COVID-19. A multivariate logistic regression analysis was performed to assess the relationship between anxiety, fear, behavioral responses, and independent variables, including exposure to news. In all, 1563 adults participated in the study. The mean age of respondents was 39.17 ± 13.5 years. Almost 55% of participants reported moderate to high-level anxiety, while fear of being affected by COVID-19 was reported 54.1%. Overall 88% reported that they had changed their behaviors to some extent. Exposure to the COVID-19 news on social media was the most influencing variable on anxiety (OR 2.21, 95% CI 1.62–3.04; P < 0.0001), fear (OR 1.95, 95% CI 1.49–2.56; P < 0.0001), and change in health behaviors (OR 2.02, 95% CI 1.28–3.19; P = 0.003) in the regression model. The fear of being infected by the COVID19 was associated with the female gender and some socioeconomic characteristics. Although exposure to the COVID-19 news on social media seemed to be associated with excess anxiety and fear, it also, to some extent, had positively changed people’s health behaviors towards preventive measures.

## Introduction

The outbreak of COVID-19 had a detrimental effect on global healthcare systems with a rapid and profound impact on every aspect of human life^1^, from the way people socialize to work, live, shop, and plan for the future^2^. In addition to the virus's global spread, another sort of pandemic developed where misleading rumors and disinformation were shared through online media, including all the influential social media and platforms such as Twitter, Facebook, Instagram, WhatsApp, and YouTube^3^. As such WHO warned all nations to not use fake information and avoid being contaminated with unfounded speculations on potential causes and cures of the disease. It was believed that sharing wrong information might have several side-effects including causing confusion, leading to risky behaviors, not following the evidence-based recommendations, and imposing psychological distress^4^. A well-known newspaper used the following title: ‘Coronavirus misinformation is dangerous. Think before you share’^4^. However, social media users, are less likely to fact-check information before sharing it^5^ and help to creating ‘infodemic’. The same story was evident during the COVID-19 pandemic and apparently even they used more social media and shared information due to isolation and quarantine^6,7^ and to receive updates about the current COVID-19 situation^8^.

Infodemic is defined as: ‘an overabundance of information—some accurate and some not—that makes it hard for people to find trustworthy sources and reliable guidance when they need it’^9^. It can intensify or lengthen the duration of pandemic^10^, and could threaten national and global efforts to control the disease outbreaks^11^.

It is well-documented that COVID-19 related misinformation increased psychological disorders among social media users^12–15^, which is a common response to any stressful situation^16^. The most common psychological consequences of pandemic related exposure to COVID-19 news on social media include anxiety disorders, depression^17^, and fear^18^. A meta-analysis of 14 cross-sectional studies indicated that spending an excessive amount of time on social media platforms was associated to a higher likelihood of experiencing symptoms of anxiety and depression^19^.

On the other hand, the responsible use of social media was reported to be associated with positive influence public awareness about the pandemic and protection against COVID-19^20^. Social media could provide users with valuable information, find solutions to problems such as uncertainties, managing crises, and help to improve emotional functioning and protect mental health^20,21^. Therefore, social networks have both positive and negative effects as a double-edged sword^22^. For instance, a higher level of fear may turn into panic, becoming dangerous and increasing harm and damage, although a certain degree of manageable fear can induce people to protect themselves and follow the measures established by states^23^.

To understand the appropriate use of social media during the COVID-19 pandemic, we must know about the consequences of exposure to social media on people’s health. A number of studies explored psychological^15,19,24–26^ and behavioral^27–29^ outcome as common and important measures. However, to the best of our knowledge, no previous study on the topic was reported from Iran. Thus, this study aimed to investigate the psychological and behavioral consequences of exposure to Covid-19 news on social media among Iranian adult population.

## Methods

### Design and participants

The present study was a telephone-based cross-sectional survey conducted during the COVID-19 pandemic in April 2020. At that time, the statistics for new COVID-19 cases were accumulating. For instance, according to the world metric records, there were about 47,593 new cases and 138 deaths in Iran on the first of April. During this period, Iran had several difficulties providing drugs and necessary supplies. However, participants were adults aged 18 and over, Iranian nationality, ability to speak in Persian, user of at least one social media platform, and experience of exposure to COVID-19 news on social media. No other restrictions were implemented. The study was conducted in accordance with the Helsinki Declaration.

### Sample size

The sample size was based on the following formula:

$${\text{n}} = {\text{Z}}^{{2}} \times {\text{P}}\;\left( {{1} - {\text{P}}} \right)/{\text{d}}^{{2}}$$

Considering Z = 1.96, P = 0.5 (assuming 50% would use social media), and d = 3% (precision), a sample size of 1067 participants was estimated. Considering the design effect of 1.5, recruiting a sample of 1600 was thought. However, in practice 1563 adults were included in the study.

### Sampling

A sample of Iranian adults aged 18 years and above were randomly selected from the list of post codes and using their mobile phones (random digit-dial). All provinces in Iran were defined as the strata and proportional to the population density of each province the required sample size was estimated for the whole country. The primary sampling unit consisted of individuals living in a given province.

### Measures

A self-designed questionnaire in Persian language consisting of two sections was administered. The items were developed based on study objectives. The first section was about socio-demographic information included the recording of age, gender, marital status, education, economic status, and occupation.

The second part of questionnaire was developed based on literature review^7,29–32^ and expert opinion. This part contained three sections (Table 1):

1. Exposure to news on the COVID-19 pandemic with three items including ‘to what extent do you follow the statistics and information on COVID-19?’, ‘to what extent do you follow formal news on COVID-19 released by the state?’ and ‘to what extent do you follow the news on COVID-19 on social media?’
2. Psychological response with two items related to anxiety and fear including ‘to what extent exposure to the news on COVID-19 made you feel anxious and worry?’, and ‘To what extent do you fear being infected with COVID-19?’
3. Behavioral response with one item ‘to what extent fear of being infected provoked you to stick to healthy behavior (hand washing, wearing face mask, social distancing)?’

**Table 1:** Exposure to news on the COVID-19 pandemic and psychological and behavioral responses.

	Not at all	Slightly	Moderately	Considerably	A great deal
Exposure to news on the COVID-19 pandemic					
To what extent do you follow the statistics and information on COVID-19?					
To what extent do you follow formal news on COVID-19 released by the state?					
To what extent do you follow the news on COVID-19 on social media?					
Psychological response					
To what extent exposure to the news on COVID-19 made you feel anxious and worry?					
To what extent do you fear being infected with COVID-19?					
Behavioral response					
a. To what extent fear of being infected provoked you to stick to hand washing?					
b. To what extent fear of being infected provoked you to stick to wearing a face mask?					
c. To what extent fear of being infected provoked you to stick to social distancing?					

Each item was rated on a 5-point Likert scale. The questionnaire was evaluated for content and face validity by seven experts (three health psychologists, two epidemiologists, and two journalists) and found to be satisfactory. The internal consistency for the questionnaire was about acceptable level (Cronbach’s alpha = 0.62).

At the begging of the phone interview, people were asked for consent. We informed the participant about the purpose of the study [exploring the association between exposure to COVID-19 news on social media and anxiety, fear, and compliance with healthy behavior]. We also explained that we are independent non-governmental research group and we are not involved with any treatment or vaccination processes. The participants were ensured about the anonymity, confidentiality and voluntary participant in the study. After they accepted to take part in the survey, Interviewers asked the questions one by one and filled in the demographic details and the six study questions. All interviewers were trained for this specific study to assure that ethical principles and consistency in data collection were considered.

### Statistical analysis

Descriptive statistics were used to report the data including mean, standard deviation, frequencies, and percentages. To assess the association between dependent variables (anxiety, fear, and self-reported behavior change) and exposure to news on COVID-19 both univariate and multivariable logistic regression analysis was performed. As such response categories for both dependent and independent variables merged to provide two classifications as follows: not at all and slightly = No and moderately, considerably, and a great deal = Yes. The results were presented as odds ratio and 95% confidence intervals. All statistical analyses were performed using R software (ver. 3.6.3, College Station, Texas, USA).

### Ethics approval and consent to participate

The study protocol including obtaining informed consent due to the COVID-19 pandemic was approved by the ethic committee of the Iranian National Institute for Medical Research Development (IR.NIMAD.REC.1399.297). All participants were made aware of the study protocol, and informed consent was obtained.

## Results

### Participants

In all 1563 adults participated in the study. The mean age of participants was 39.17 ± 13.5 years. Most participants had secondary (60.1%) or higher education (35.3%), half of the sample were had intermediate economic status, while the vast majority of individuals were high school-educated adults (60.1%) and university or college-leveled institutes (35.3%). The description of sociodemographic variables is summarized in Table 2.

**Table 2:** Characteristics of Iranian adults who participated in the study (n = 1563).

Variables	No. (%)
Age (Mean ± SD)	39.17 ± 13.5
Gender	
Female	775 (49.6)
Male	788 (50.4)
Marital status	
Single	342 (21.9)
Married	1160 (74.3)
Divorced/widowed	61 (3.8)
Economic status (n = 1313)	
Poor	322 (24.5)
Intermediate	619 (47.2)
Good	372 (28.3)
Education (n = 1013)	
Primary	74 (4.7)
Secondary	428 (27.3)
Higher	511 (32.7)
Employment status (n = 1453)	
Unemployed	64 (4.4)
Housewife	516 (35.5)
Student	95 (6.5)
Employed	687 (47.3)
Retired	91 (6.3)

### Descriptive findings

Moderate to high-level of anxiety was reported by 55.4% of participants and this was 54.1% for fear of being affected by coronavirus disease. Eighty-eight percent of people reported that they have changed their behaviors. The detailed results are shown in Table 3.

**Table 3:** Descriptive statistics for the study measures.

	Not at all	Slightly	Moderately	Considerably	A great deal
	No. (%)	No. (%)	No. (%)	No. (%)	No. (%)
To what extent do you follow the statistics and information on COVID-19? (n = 1559)	70 (4.5)	307 (19.7)	342 (21.9)	539 (34.6)	301 (19.3)
To what extent do you follow formal news on COVID-19 released by the state? (n = 1560)	153 (9.8)	447 (28.7)	306 (19.6)	477 (30.6)	177 (11.3)
To what extent do you follow the news on COVID-19 on social media? (n = 1561)	517 (33.1)	379 (24.3)	204 (13.1)	340 (21.8)	121 (7.8)
To what extent exposure to the news on COVID-19 made you feel anxious and worry? (n = 1044)	137 (13.1)	328 (31.4)	211 (20.2)	261 (25.0)	107 (10.2)
To what extent do you fear being infected with COVID-19? (n = 1561)	375 (24.0)	342 (21.9)	268 (17.2)	324 (20.8)	252 (16.1)
To what extent fear of being infected provoked you to stick to healthy behaviors? (n = 1188)	29 (2.4)	113 (9.5)	136 (11.4)	504 (42.4)	406 (34.2)

### Feeling of anxiety

In the multivariable logistic regression model, experience of anxiety significantly was associated with exposure to news on social media (OR 2.21, 95% CI 1.62–3.04; P < 0.0001). The results are shown in Table 4.

**Table 4:** The results obtained from logistic regression analysis for feeling anxiety.

	Univariate analysis		Multivariate analysis	
	OR (95% CI)	P-value	OR (95% CI)	P-value
Age	0.99 (0.98–1.01)	0.68	0.99 (0.97–1.06)	0.43
Gender				
Female	1.0 (ref.)		1.0 (ref.)	
Male	1.33 (1.03–1.69)	**0.024**	1.44 (0.93–2.24)	0.10
Marital status				
Single	1.0 (ref.)		1.0 (ref.)	
Married	1.05 (0.79–1.39)	0.71	0.85 (0.54,1.34)	0.48
Divorced/widowed	1.33 (0.62–2.85)	0.46	1.32 (0.45–3.9)	0.61
Economic status				
Very good	1.0 (ref.)		1.0 (ref.)	
Intermediate	0.82 (0.61–1.1)	0.18	0.94 (0.61–1.45)	0.79
Poor	0.93 (0.83–0.36)	0.66	0.95 (0.57–1.56)	0.83
Education				
Primary	1.0 (ref.)		1.0 (ref.)	
Secondary	0.27 (0.06–1.35)	**0.008**	0.74 (0.36–1.55)	0.42
Higher	0.25 (0.24,0.74)	**0.003**	0.56 (0.26–1.21)	0.14
Employment status				
Unemployed	1.0 (ref.)		1.0 (ref.)	
Housewife	0.74 (0.41–1.36)	0.34	0.77 (0.32–1.85)	0.56
Student	1.15 (0.62–2.14)	0.65	0.65 (0.25–1.72)	0.56
Employed	0.73 (0.35–1.51)	0.4	0.72 (0.33–1.57)	0.41
Retired	0.43 (0.19–0.98)	**0.046**	0.47 (0.15–1.4)	0.18
Exposure to information and statistics on COVID-19				
No	1.0 (ref.)		1.0 (ref.)	
Yes	1.05 (0.75–1.45)	0.78	0.94 (0.57,1.55)	0.83
Exposure to formal news about COVID-19				
No	1.0 (ref.)		1.0 (ref.)	
Yes	1.4 (1.04–1.85)	**0.02**	1.4 (0.98–2.1)	0.059
Exposure to COVID-19 news on social media				
No	1.0 (ref.)		1.0 (ref.)	
Yes	2.31 (1.78–2.98)	**0.0001**	2.21 (1.62–3.04)	**0.0001**

*Bold values are significant.

### Feeling of fear

Being female (OR 2.17, 95% CI 1.46–3.22; p-value < 0.001), intermediate economic status (OR 0.73, 95% CI 0.51–0.99; p-value = 0.049), being employed (OR 2.05, 95% CI 1.01–6.22; p-value = 0.047), higher exposure to information and statistics on COVID-19 (OR 1.52, 95% CI 1.1–2.12; p-value = 0.011), exposure to formal news on COVID-19 (OR 1.62, 95% CI 1.20–2.20; p-value = 0.002) and exposure to social media for updating on the COVID-19 news (OR 1.95, 95% CI 1.49–2.56; p-value < 0.001) showed significant association with feeling of fear. The results are presented in Table 5.

**Table 5:** The results obtained from logistic regression analysis for feeling of fear.

Characteristics	Univariate analysis		Multivariate analysis	
	OR (95% CI)	P-value	OR (95% CI)	P-value
Age	0.99 (0.98–1.01)	0.08	0.99 (0.98–1.05)	0.25
Gender				
Male	1.0 (ref.)		1.0 (ref.)	
Female	2.04 (1.67–2.5)	** < 0.0001**	2.17 (1.46–3.22)	** < 0.0001**
Marital status				
Single	1.0 (ref.)		1.0 (ref.)	
Married	1.14 (0.89–1.45)	0.29	1.22 (0.82–3.83)	0.32
Divorced/widowed	1.64 (0.94–2.9)	0.08	1.79 (0.84–3.83)	0.21
Economic status				
Very good	1.0 (ref.)		1.0 (ref.)	
Intermediate	0.75 (0.57–0.99)	**0.042**	0.73 (0.51–0.99)	**0.049**
Poor	0.86 (0.63–1.62)	0.32	0.89 (0.6–1.32)	0.57
Education				
Primary	(ref.)		1.0 (ref.)	
Secondary	0.87 (0.65–1.16)	0.33	0.9 (0.64–1.49)	0.91
Higher	1.06 (0.79–1.42)	0.69	1.02 (0.62–1.65)	0.93
Employment status				
Unemployed	1.0 (ref.)		1.0 (ref.)	
Employed	1.54 (0.92–2.61)	0.1	2.05 (1.01–6.22)	**0.047**
Housewife	2.56 (1.5–4.36)	**0.001**	1.9 (0.88–4.13)	0.10
Student	1.96 (1.03–3.75)	**0.04**	1.76 (0.74–4.23)	0.20
Retired	1.7 (0.89–3.27)	0.11	2.2 (0.97–4.99)	0.058
Exposure to information and statistics on COVID-19				
No	1.0 (ref.)		1.0 (ref.)	
Yes	1.66 (1.26–2.15)	**0.0001**	1.52 (1.1–2.12)	**0.011**
Exposure to formal news about COVID-19				
No	1.0 (ref.)		1.0 (ref.)	
Yes	1.86 (1.47–2.36)	**0.0001**	1.62 (1.20–2.20)	**0.002**
Exposure to COVID-19 news on social media				
No	1.0 (ref.)		1.0 (ref.)	
Yes	2.003 (1.63–2.46)	**0.0001**	1.95 (1.49–2.56)	** < 0.0001**

*Bold values are significant.

### Self-reported behavioral responses

The only factor that influenced behavior change was exposure to the COVID-19 news on social media (OR 2.02, 95% CI 1.28–3.19; P = 0.003). In fact, people reported that they took more preventive measures (hand washing, wearing face mask, social distancing) after exposure to COVID-19 news on social media. The results are reported in Table 6.

**Table 6:** The results obtained from logistic regression for self-reported behavior change.

	Univariate analysis		Multivariate analysis	
	OR (95% CI)	P-value	OR (95% CI)	P-value
Age	0.99 (0.98–1.01)	0.44	0.99 (0.97–1.02)	0.79
Gender				
Female	1.0 (ref.)		1.0 (ref.)	
Male	1.6 (1.12–2.28)	**0.009**	1.86 (0.97–3.54)	0.059
Marriage status				
Single	1.0 (ref.)		1.0 (ref.)	
Married	1.19 (0.79–1.78)	0.41	1.15 (0.6,2.20)	0.67
Divorced/widow	1.24 (0.46–3.35)	0.67	1.01 (0.26,3.82)	0.98
Economic status				
Very good	1.0 (ref.)		1.0 (ref.)	
Intermediate	0.78 (0.51–1.18)	0.24	1.31 (0.78,2.41)	0.26
Poor	0.49 (0.18–1.36)	0.17	0.91 (0.48,1.68)	0.75
Education				
Primary	1.0 (ref.)		1.0 (ref.)	
Secondary	1.07 (0.64–1.8)	0.77	0.74 (0.35,1.57)	0.43
Higher	0.86 (0.59–1.7)	0.97	0.68 (0.29,1.58)	0.37
Employment status				
Unemployed	1.0 (ref.)		1.0 (ref.)	
Housewife	2.39 (1.17–4.86)	**0.016**	2.16 (0.69–6.7)	0.18
Student	3.75 (1.79–7.86)	** < 0.0001**	2.12 (0.61–7.38)	0.23
Employed	2.65 (1.04–6.75)	**0.04**	2.44 (0.90–6.59)	0.07
Retired	0.43 (0.69–4.6)	0.23	1.26 (0.33–4.83)	0.73
Exposure to information and statistics on COVID-19				
No	1.0 (ref.)		1.0 (ref.)	
Yes	1.4 (0.91–2.16)	0.12	1.33 (0.79–2.558)	0.28
Exposure to formal news about COVID-19				
No	1.0 (ref.)		1.0 (ref.)	
Yes	1.93 (1.32–2.82)	**0.01**	1.44 (0.89–2.32)	0.13
Exposure to COVID-19 news on social media				
No	1.0 (ref.)		1.0 (ref.)	
Yes	2.11 (1.45–3.07)	**0.0001**	2.02 (1.28–3.19)	**0.003**

## Discussion

**Figure 1:**
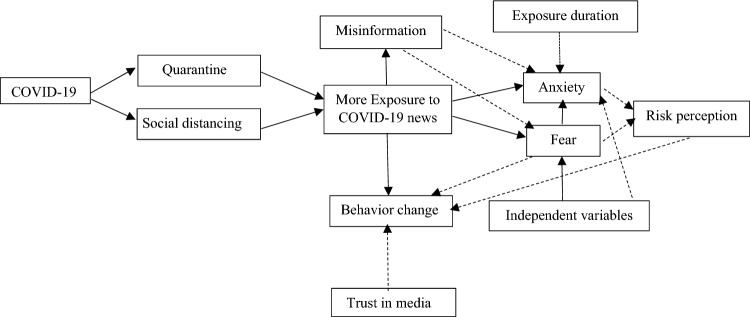
A schematic view of the mechanism of exposure to the COVID-19 news on social media.

The media play a crucial role in response to crises by informing the public, making positive behavioral changes, and affecting mental health and well-being^33^. This study reported that exposure to the COVID-19 news on social media induced anxiety and fear, and also it showed some positive changes among participants. A schematic view of the mechanism of such observation is provided in (Fig. 1). This was proposed from the study findings, and from what one could find in the literature^24,34,35^.

### Exposure to COVID-19 news

During the pandemic of COVID-19, people tend to use the social media more often^36^. Perhaps spending more time on social media during the COVID-19 pandemic could be due to two major reasons: quarantine and physical/social distancing (isolation, in-home lockdown, closure of services and public spaces, and loneliness)^37^. One might argue that these factors contributed to the increased use of social media. In addition, during pandemic social media was a major source for communication between families and friends. Even the use of social media for educational activity or office works contributed to extra use of the social media for news and views.

### Anxiety

The finding showed that more exposure to the news on COVID-19 on social media was associated with greater anxiety. Evidence suggests that more access to information on social media could be stressful and induce more anxiety^19,38,39^. A study from Iran confirmed that online news played a critical role in COVID-19 anxiety^40^. A cross-sectional study conducted in China reported similar results, where more exposure to news on social media was significantly associated with greater anxiety^7^. Contrary to the majority published papers, a study conducted in Romania revealed that depression and anxiety were not associated with exposure to information regarding COVID-19^41^. Possible explanations for this may include differences in measures used or might be due t o cultural or socioeconomic differences. However, when individuals read the news and cannot do anything to prevent or reduce the risk of the disease, they begin to see themselves as vulnerable, and anxiety emerges. One other possible explanation is the fact that at the time the study commenced, the nature of COVID-19 was unknown, and thus it seemed a scary phenomenon and induced anxiety, fear and uncetainties. As such, one might argue that it is essential to see when and how psychological distress, including anxiety, depression, distress, or fear, is measured.

The current study did not assess the possible relationship between anxiety and exposure duration. Evidence suggests that more exposure to social media was associated with more psychological distress about the virus^24^. A study showed that more than four hours of using social media was related to a higher level of anxiety^42^. One argument is that more exposure to social media leads to more exposure to fake news and misinformation.

### Fear

The current study showed that exposure to news on social media was related to higher levels of fear. This leads us to believe that social media exposure could be an indicator of even other negative emotions. Similar findings have been reported in other investigations in various settings^15,25,26,43,44^. For instance, a report from Hong Kong revealed that social media provoked fear in society^45^. The current study was conducted at the beginning of the COVID-19 pandemic when social media was full of negative news such as high daily statistics of the disease and deaths. Besides, social media users were facing a massive amount of information, where most of them did not have enough knowledge and health literacy to distinguish true information from fake news. Furthermore, usually, the governments also did not have an effective strategy to manage this situation. Thus, combining the above factors led to an increased fear among users. Experiencing fear and its association with positive preventive behaviors during the COVID-19 pandemic has been reported previously in several studies^15,36,44,46^. Although the current study did not assess the possible relationship between fear and preventive behaviors, it seems that the implementation of educational interventions, including mass media campaigns such as ‘Together we will beat the covid-19’, might explain why people took preventive behaviors while they were frightened.

### Psychological factors and independent variables

The current study did not show a significant relationship between most independent variables and anxiety. However, a study from Iran reported that anxiety was associated with female gender, younger age, and experience of the COVID-19 among family members or friends^47^. Similarly, a study reported that psychological factors were associated with being female, having cardiovascular diseases, smoking, and having a history of the COVID-19 symptoms, including fever, cough, and shortness of breath^48^. The role of independent variables in anxiety is undeniable.

We found that different factors, including female gender, intermediate economic status, being employed, following the COVID-19 statistics and formal news released by the state, and exposure to news on social media, had a significant relationship with fear. A study showed that COVID-19 has significantly affected people's fear due to incidents like economic slowdown, loss of jobs, losing loved ones, and so on^49^. Perhaps such observation also was true for the current study where due to economic sanction and some limitations for providing vaccine supply those who followed news on social media were more likely to experience ore fear as expected.

### Behavioral responses

The findings showed that exposure to social media could positively influence health behaviors related to COVID-19 prevention. Similarly, some studies have demonstrated that frequent social media exposure regarding COVID-19 was associated with adopting preventive measures (e.g., face mask-wearing and handwashing)^20,27,29^. An online survey among American people showed that news monitoring was associated with greater social responsibility, more disinfecting, and greater caution about the severity of COVID-19^50^. It might be the result of the efforts of official departments to increase the public’s awareness of prevention strategies by providing updated information about COVID-19 on websites and social media^51^. According to behavioral models, exposure to social media increasing the users' awareness about how protecting themselves against COVI-19. Therefore, besides increases the perceived threat, it is a cue to action that encourages individuals to change their behaviors. So, the effect of social media on individuals’ protective behaviors can be influenced by different factors such as the type of information that users are exposed to, the level of the perceived threat, and the self-efficacy of individuals to copping the stress and manage the control of risk.

### Exposure to COVID-19 misinformation

A number of social media users produce, release and transfer information that may lead to the dissemination of misinformation on social media^52,53^. So social media news often contains widespread misinformation, fake news and rumors^54^, that may cause many users psychological problems^55^. By analyzing the phenomenon of fake news in health, it was observed that false information could cause psychological disorders, panic, fear, depression, and fatigue^14^. For instance, one study showed that fear of COVID-19 and misunderstanding were associated with problematic social media usage, which led to direct or indirect psychological distress and insomnia^13^. Thus, the governments should consider the adverse consequence of misinformation during the COVID-19 pandemic on people's mental health^47^ and implment appropirate interventions. In such situations, the existence of the 'infodemics' team is necessary to deliver right information to the right people or a broader public audiences.

### Risk perception

Risk perception is an important component of behavioral change^56^. According to the health behavioral models, information provides cues that influence perceptions regarding health threats^35,57^. According to the extended parallel process model (EPPM) as one of the relevant behavioral change models, individuals undergo two cognitive appraisals during exposure to a risky situation: the ability to respond to the recommended message (efficacy) and the perceived threat^24^. When the threat of COVID-19 is high and efficacy is low, people usually act to protect themselves from the fear rather than the danger itself^58^. A study showed that fear was positively associated with forming risk perceptions during an outbreak^36,59,60^. Individuals utilize psychological defense strategies to manage their fears in this situation^57^. A number of studies showed that when individuals obtain information from social media about COVID-19, they may perceive COVID-19 as a health threat and experience subsequent anxiety, depression, and fear^61^. Conversely, when perceived efficacy is high, people usually are motivated to protect themselves from danger and might follow the recommended massages^58^. In this regard, risk perception is related to adopting preventive behaviors such as social distancing and mask use^36,60^. Finding of a previous study revealed that self-efficacy was significantly associated with trust in government and media information on the pandemic^7^. Therefore, producing appropriate and reliable information would be necessary.

### Strengths and limitations

Although the study benefited from a relatively good sample size and was selected based on a random sampling method, generalizing the findings might be challenging. This was a cross-sectional study in nature and thus could not indicate causality, and the findings should be interpreted with caution. People with mental health disorders might experience higher fear and anxiety regardless of social media exposure. Since we did not collected information in this regard, this should be considered as limitation. This study was conducted at the start of the COVID-19 pandemic, and the anxiety, fear, and behavioral responses might have been influenced by the novelty and uncertainty of the situation rather than social media exposure *pre se*. Thus the findings should be interpreted with caution. We did not ask participants how much time they were spending on social media. Also, we did not explore ‘infodemic’ and how much did misinformation contributed to fear, anxiety, and behavioral responses. This could be a significant factor that study has missed. It is recommending that a such variable be investigated in future studies. Our study did not distinguish between social media platforms. Different platforms may induce different levels of fear, anxiety, and behavioral responses due to their varied ways of information dissemination, user demographics, and misinformation controls.

## Conclusion

The findings demonstrated that exposure to the COVID-19 news on social media was associated with increased anxiety and fear. Yet, it might bring some positive behavioral changes. Therefore, improving people's media literacy in order to make them be able in identifying trusted information and share reliable content on social media seems necessary. Also, the governments should deal with 'infodemic' by providing timely up-to-dated and reliable information to prevent spread of misinformation. They are also responsible to introduce credible sources for reliable information.

## Data Availability

The datasets used and analyzed are available from the corresponding author on reasonable request.
